# Draft Genome Sequences of Nine Cyanobacterial Strains from Diverse Habitats

**DOI:** 10.1128/genomeA.01676-16

**Published:** 2017-03-02

**Authors:** Tao Zhu, Shengwei Hou, Xuefeng Lu, Wolfgang R. Hess

**Affiliations:** a Key Laboratory of Biofuels, Shandong Provincial Key Laboratory of Synthetic Biology, Qingdao Institute of Bioenergy and Bioprocess Technology, Chinese Academy of Sciences, Beijing, China; b Genetics and Experimental Bioinformatics, Faculty of Biology, University of Freiburg, Freiburg im Breisgau, Germany

## Abstract

Here, we report the annotated draft genome sequences of nine different cyanobacteria, which were originally collected from different habitats, including hot springs, terrestrial, freshwater, and marine environments, and cover four of the five morphological subsections of cyanobacteria.

## GENOME ANNOUNCEMENT

Cyanobacteria are oxygenic photosynthetic prokaryotes that can be found at diverse geographical and ecological locations (1). Based on morphological criteria, cyanobacteria can be divided into five subsections, I to V (2). Initially, a large number of publicly available genome sequences were obtained from subsection I strains, but more recent attempts have been focusing on taxa without representative genome sequences (3). In terms of both fundamental research and biotechnological applications, improved genomic coverage would be advantageous for certain lineages. In this study, nine cyanobacterial strains were sequenced, including three hot spring strains, *Chroogloeocystis siderophila* NIES-1031 (subsection II, originally collected from bottom mud of LaDuke Hot Springs, MT, USA), *Hydrococcus rivularis* NIES-593, and *Fischerella major* NIES-592 (subsections II and V, respectively, originally collected from Yukawa Hot Spring, Japan). Three strains were of terrestrial origin: *Nostoc calcicola* FACHB-389 (subsection IV, originally collected from soil in Utrecht, The Netherlands), *Calothrix* sp. NIES-2101, and *Scytonema* sp. NIES-2130 (both subsection IV and originally collected from the University of Hyogo, Himeji, Japan). Two strains belonging to subsection III were originally collected from freshwater, *Leptolyngbya* sp. NIES-30 from a paddy field in Akita, Japan, and *Phormidium ambiguum* NIES-2119 from a pond in northeast Brazil, whereas *Oscillatoria rosea* NIES-208 (subsection III), a marine isolate, was originally collected from Asaji Bay, Mitsushima, Japan. All strains were cultured in 500-ml flasks containing 300 ml of medium, bubbled with sterile air, and illuminated with 30 to 50 μmol photons m^-2^ s^-1^ white light in medium BG11 (4), except for *Oscillatoria rosea* NIES-208, which was cultivated in A^+^ medium (5).

Genomic DNA was extracted from exponential-growth phase cells using the EZ-10 plant genomic DNA purification kit (Sangon Biotech, China). Extracted genomic DNA of *Oscillatoria rosea* NIES-208, *Nostoc calcicola* FACHB-389, *Fischerella major* NIES-592, and *Hydrococcus rivularis* NIES-593 was sheared to ~500-bp fragments and then sequenced using the paired-end protocol of the Illumina HiSeq 2000 system (2 × 100 bp). The other five strains were sequenced with a fragment size of 300 to 500 bp using the paired-end protocol of Illumina MiSeq (2 × 300 bp). Adapter sequences were removed and low-quality ends trimmed using Trimmomatic version 0.33 (6), with a minimum Phred score of 20 in a sliding window of 4. Reads >20 nucleotides (nt) were used for *de novo* assembly using SPAdes version 3.9.0 (7) in “--meta” mode with default parameters. Contigs >2 kb were binned using MaxBin version 2.2.1 (8), and the completeness and contamination were assessed using CheckM version 1.0.5 (9). Contigs binned to *Cyanobacteria* were scaffolded using BESST version 2.2.4 (https://github.com/ksahlin/BESST) and FinishM version 0.0.9 (https://github.com/wwood/finishm) and then polished using Pilon version 1.20 (10). Scaffolds were taxonomically classified using Kaiju (11) and PhyloPythiaS+ (12). Those not assigned to *Cyanobacteria* were manually checked using BLASTN (13), and contaminants were removed. The final assemblies were annotated using the NCBI PGAAP (14).

### Accession number(s).

The draft genome sequences of the nine cyanobacterial strains have been deposited as NCBI whole-genome shotgun (WGS) projects at DDBJ/EMBL/GenBank under the accession numbers listed in Table 1; the versions described in this paper are the first versions.

**TABLE 1:** Genome features and GenBank accession numbers of the strains

Strain	Habitat	Biosample no.	Accession no.	Genome size (Mb)	Coverage (×)
*Oscillatoria rosea* NIES-208	Marine	SAMN05890674	MRBY00000000	4.0	102
*Nostoc calcicola* FACHB-389	Terrestrial	SAMN05890684	MRBZ00000000	8.8	45
*Fischerella major* NIES-592	Hot spring	SAMN05890685	MRCA00000000	5.5	156
*Hydrococcus rivularis* NIES-593	Hot spring	SAMN05890686	MRCB00000000	5.0	136
*Chroogloeocystis* *siderophila* NIES-1031	Hot spring	SAMN05890687	MRCC00000000	4.9	56
*Calothrix* sp. NIES-2101	Terrestrial	SAMN05890688	MRCD00000000	9.7	13
*Phormidium ambiguum* NIES-2119	Freshwater	SAMN05890689	MRCE00000000	7.2	117
*Scytonema* sp. NIES-2130	Terrestrial	SAMN05890690	MRCF00000000	9.3	44
*Phormidium tenue* NIES-30	Freshwater	SAMN05890691	MRCG00000000	5.7	84
